# Editorial: Vascular wall markers by imaging: from physiology to pathological features

**DOI:** 10.3389/fphys.2023.1196962

**Published:** 2023-04-24

**Authors:** Guillaume Goudot, Jean-Michel Davaine

**Affiliations:** ^1^ INSERM U970 PARCC, Université Paris Cité, Paris, France; ^2^ Department of Vascular Surgery, Sorbonne Université, Paris, France

**Keywords:** artery, microcirculation, endothelial cell, imaging, stiffness

The vascular wall is a critical part of the cardiovascular system and undergoes constant remodeling. Most so-called cardiovascular events originate as a direct consequence of a wall injury. Given the various layers of the vascular wall, the numerous interactions between the different cell types (first and foremost being endothelial cells and vascular smooth muscle cells), and the great diversity of wall types depending on the vascular bed, the study of the wall is complex but promising in developing an understanding of pathophysiology and in the development of targeted therapies for cardiovascular diseases. The contribution of imaging to the study of the vascular wall *in vivo* is essential. Technological development enables constant progress in this field. In addition, understanding the mechanisms underlying arterial stiffness and improving assessment of this is central to cardiovascular research as well as clinical routine. Evaluation of central arterial stiffness all the way to the distal vessels through new imaging modalities is as challenging as it is insightful. This Research Topic aimed to collect original works in this field by gathering contributions from various fields of expertise in imaging techniques, such as magnetic resonance imaging (MRI), computed tomography (CT), ultrasound, or optical imaging, and types of application (type of vessels and pathology explored).

Svensson et al. assessed the case of Takayasu’s arteritis based on several parameters: an analysis of arterial stiffness through evaluation of the augmentation index, measured on the radial artery; and, more surprisingly, an evaluation of the consequences of the disease for microcirculation through measurement of the microcirculatory peak oxygen saturation in the skin after induced ischemia with laser Doppler flowmetry. Their work reflects the possibilities of assessment techniques that combine measurements related to the wall of the main elastic arteries with an assessment of microcirculation, which is still difficult to achieve through imaging.

Weismann et al. also followed a multi-parameter approach to assess arterial fragility in Marfan’s disease. Although the rate of aortic dissection is particularly high in this condition, developing risk markers using imaging, beyond simple measurement of arterial diameter, is challenging. The authors identify markers of arterial stiffness (distensibility, augmentation index, and pulse wave velocity), markers of endothelial function according to the reactive hyperemia index, and biological markers. Although markers of arterial stiffness are associated with greater wall damage and aneurysmal growth, these markers are not currently validated in clinical practice. At the individual level, risk prediction remains very limited. Although these findings are yet to be evaluated prospectively, multiparametric assessments might achieve the goal of better predicting vascular events.

Kralj et al. offer an instructive review of Wavelet analysis of laser Doppler, including its technical aspects and applications in studying endothelial function. Imaging microvessels *in vivo* remains particularly complex. Indirect techniques such as Wavelet analysis of laser Doppler flowmetry play an important and expanding role in evaluating microvascular dysfunction.

In an elegant piece of work, Kiema M et al. assess the importance of wall shear stress (WSS) in media remodeling. WSS of the ascending aorta is now accessible via 4D MRI. Shear stress related to blood flow plays an important biological role, affecting endothelial cells transmitted to the entire wall. Thus, alterations in the media, such as elastic fiber content, are influenced by local variations in WSS. Without constituting a direct marker of the arterial wall, assessment of WSS shows the importance of coupling wall imaging to hemodynamic assessment to better appreciate dynamic modifications to the wall in relation to its immediate environment. In terms of risk assessment, WSS is indeed a predictive marker for dilatation in the case of bicuspid aortic valve [1].

Lastly, Goudot et al. have developed a method to track deformation of the aortic wall, this time using ultrafast ultrasound imaging. The use of high-frame-rate plane-wave imaging provides tissue Doppler visualization over the entire 2D image and tracking of ascending aortic deformation to obtain parameters related to wall stiffness, a marker of early remodeling. Applications relating to changes in the ascending aorta associated with a bicuspid aortic valve are an example of a possible assessment of segmental aortic impairment that could also be applied to more general cases of risk for aortic aneurysm.

Taken together, the aforementioned articles showcase a wide spectrum of new experimental methods for the measurement and analysis of multimodal data on vascular wall involvement in cardiovascular physiology and pathology.

